# Commentary: Why sprint interval training is inappropriate for a largely sedentary population

**DOI:** 10.3389/fpsyg.2017.01603

**Published:** 2017-09-13

**Authors:** Jennifer Robertson-Wilson, Mark Eys, Tom J. Hazell

**Affiliations:** Department of Kinesiology and Physical Education, Wilfrid Laurier University Waterloo, ON, Canada

**Keywords:** high-intensity interval training, exercise, affect, enjoyment, replication, measurement

Hardcastle et al. (2014) argued that an inactive population is unlikely to engage in sprint interval training (SIT) due to poor affective responses, low self-efficacy and motivation, and increased challenges to self-regulation. Their opinion article offers reasonable critiques to the potential broader effectiveness of SIT vis-a-vis the efficacy demonstrated within laboratory trials. However, three commentary responses (Del Vecchio et al., 2015; Astorino and Thum, 2016; Jung et al., 2016) have since been published with one common thread being to question the assumption that low affective perceptions necessarily accompany engagement in SIT. We have followed this debate with interest, in particular regarding affective responses to SIT [and, more broadly, high-intensity interval training (HIIT)]^1^. Building on the current debate, we further propose that in order to advance the research agenda, and specifically our understanding of “the acceptability of, and affective responses to, SIT programs” (Hardcastle et al., 2014, p. 2), a discussion of how and when affective responses are *measured* is warranted.

Affect is believed to be related to, but distinct from, both emotion and mood (Ekkekakis, 2013). Ekkekakis (2013) further noted that “[e]xamples of core affect include pleasure, displeasure, tension, calmness, energy, and tiredness. A person experiences core affect constantly, although the nature and intensity of affect varies over time” (p. 38). The relationship between affect (and other psychological responses) and engagement in HIIT/SIT was examined in a recent scoping review (Stork et al., 2017). The review authors identified inconsistencies in the *timing* of when measures of affect were given across the included 42 studies, which resulted in some difficulty for comparisons across studies (Stork et al., 2017). In addition to timing, they also observed that “[m]any different measures were used to evaluate the same psychological constructs” (Stork et al., 2017, p. 25). It is this heterogeneity in measures we suggest requires further discussion and more purposeful consideration in future studies, in particular as it relates to replication.

In the 17 studies that measured affect, Stork et al. (2017) reported that six different measures were used with the most common measure of affect being the Feeling Scale (FS; Hardy and Rejeski, 1989). Another psychological indicator, enjoyment, was assessed in 22 studies with five different measures. Stork et al. reported that the Physical Activity Enjoyment Scale (PACES; Kenzierski and DiCarlo, 1991) was most often used. Given that one of the key concerns for HIIT/SIT is negative affective responses when faced with arduous physical activity of this nature, further consideration should be given to which measures of affect are adopted, the accuracy of those measures, and their consistency of use among researchers. This is especially important for study replication and comparison. For example, in our own work (Townsend et al., 2017), we consciously selected the same affective instruments used by Jung et al. (2014) in order to draw comparisons between the two studies. Although Ekkekakis (2013) cautions against selecting a measure with the sole rationale being past practice or consistency with previous research, there is some worth in *using appropriate measures consistently* to allow for a body of literature to develop. In fact, Decker and Ekkekakis (2017) also used the PACES and FS in their own research, consistent with Jung et al. and Townsend et al., which does allow for discussion pertaining to the merits of different protocols. This is certainly preferable to comparing across a variety of measures such as the Subjective Exercise Experiences Scale (used by Tritter et al., 2013) and Self-Assessment Manikin (used by Saanijoki et al., 2015).

To advance the research agenda in determining the uptake of HIIT/SIT in the population as related to affective experiences, and in line with Ekkekakis (2013), we first recommend that researchers should (if they do not already) ask themselves *what affect instrument is most appropriate for the current study and why*? We argue that replication must be part of this rationale. Second, given the opposing viewpoints pertaining to the potential effectiveness of HIIT/SIT and the need to replicate and verify study findings, we suggest that researchers consider pre-registering their studies. Pre-registration, including both reviewed and unreviewed approaches, allows for methods and hypotheses to be timestamped prior to the actual execution of the study. van't Veer and Giner-Sorolla (2016) highlighted that pre-registration can serve to put theory and methods to the forefront, reduce publication bias (e.g., a tendency to only publish significant findings), and decrease reporting bias (e.g., full disclosure of results instead of picking specific findings that fit the researcher's narrative). In the present debate, pre-registration might be a useful process to encourage in-depth theoretical exposition (e.g., exposing the nuances of affect and emotion as they pertain to HIIT/SIT), consistent measurement choices, and greater confidence in the findings. Also, van't Veer and Giner-Sorolla noted that pre-registration is useful in situations where there are opportunities for “adversarial collaboration between scholars with opposing views” (p. 5), which may be the case for researchers examining the relative effectiveness of these types of exercise regimens. Finally, we also advocate for interdisciplinary research teams. Our research team has elected to broach the SIT research agenda using an “adversarial collaboration” through two (exercise psychology and exercise physiology) interdisciplinary perspectives. It is clear from the current debate that not all researchers in the field buy into the potential effectiveness of SIT. Greater consistency across exercise protocols, measurement timing (Stork et al., 2017), and psychological assessments will serve to clarify the populations for whom and conditions under which SIT may work in the real world.

## Author contributions

All authors contributed to the conceptualization of the manuscript. JRW drafted the manuscript with ME and TJH providing substantive additions and edits. All authors approved to final manuscript.

### Conflict of interest statement

The authors declare that the research was conducted in the absence of any commercial or financial relationships that could be construed as a potential conflict of interest.

## Footnotes

^1^HIIT uses short bouts of exercise at or near maximal intensities (approx. 80–100% of maximal heart rate) separated by recovery periods. SIT represents a more intense variation of HIIT and includes brief bouts of supramaximal exercise (at or above 100% VO_2max_) also separated by recovery periods.
